# Revisiting morphine-augmented hepatobiliary imaging for diagnosing acute cholecystitis: the potential pitfall of high false positive rate

**DOI:** 10.1007/s00261-013-0067-8

**Published:** 2014-01-09

**Authors:** Bor-Tau Hung, Katie S. Traylor, Ching-yee Oliver Wong

**Affiliations:** 1 Department of Diagnostic Radiology and Molecular Imaging, Oakland University William Beaumont School of Medicine, 3601 W. 13 Mile Road, Royal Oak, MI 48073 USA; 2 Department of Nuclear Medicine, Kaohsiung Chang Gung Memorial Hospital and Chang Gung University College of Medicine, Kaohsiung, Taiwan

**Keywords:** Morphine, Hepatobiliary imaging, False positive, Acute cholecystitis

## Abstract

**Purpose:**

The aim of this retrospective study was to investigate the efficacy of morphine-augmented hepatobiliary imaging (MAHBI) for diagnosing acute cholecystitis (AC).

**Methods:**

Sixty-eight patients (Male:Female = 36:32, age = 54 ± 17 years) referred for diagnosis of AC by 30-min post-morphine MAHBI after the standard 1-h imaging were recruited. Non-visualization of gallbladder on 30-min post-morphine images by visual analysis was considered positive. Final diagnosis of pathological examination for all patients was used as the gold standard.

**Results:**

There was significant correlation of AC and MAHBI (*p* < 0.05). There were 45 true positive (TP), 19 false positive (FP), 4 true negative (TN), and no false negative (FN) cases using gallbladder visualization by 30-min post-morphine as the criteria, with a high false positive rate of 83%. The sensitivity, specificity, accuracy, positive and negative predictive values of MAHBI in detecting AC were 100%, 17%, 72%, 70%, and 100%, respectively.

**Conclusions:**

MAHBI is sensitive but may not specific for diagnosing AC due to the potential pitfall of high false positive rate. Correlation with other clinical findings is recommended for optimal patient management.

Acute cholecystitis (AC) is an acute inflammation in the gallbladder. Acute calculous cholecystitis (ACC) always starts with persistent obstruction of gallbladder outlet by a stone impacted in the neck of the gallbladder, Hartmann’s pouch, or the cystic duct. As a result, pressure inside the gallbladder rises, causing rapid distention of the gallbladder, decreased blood supply, ischemia of the gallbladder walls, bacterial invasion with acute inflammation, and possible perforation. Gallstones are not found in 5% to 10% of patients with acute cholecystitis (acute acalculous cholecystitis). The pathogenesis of acute acalculous cholecystitis (AAC) is not fully understood and probably involves ischemia, biliary stasis with activation of local bacterial flora, and chemical inflammation [1]. Repeated episodes of acute inflammation may lead to chronic cholecystitis (CC). Gallbladder walls will become thick, infiltrated with inflammatory cells. The subsequent development of fibrosis leads to a loss of contractility of the gallbladder. Hepatobiliary imaging is considered the procedure of choice for diagnosing AC. Diagnosis of AC is made when the gallbladder is not seen for up to 4 h. Morphine-augmentation has been used as an alternative to delayed imaging to shorten the total imaging time required to diagnose AC [2]. The entire study can be terminated in 90 min in contrast to the 4 h or more with delayed imaging. Previous studies of patients with suspected AC have indicated that the morphine-augmentation is as useful as, or more useful than, delayed imaging [2–7]. However, morphine-augmented hepatobiliary imaging (MAHBI) as well as traditional delayed imaging are susceptible to false positive results which, in turn, result in decreased specificity [5, 8–10]. Prolonging post-morphine imaging to reduce false positive rate has recently been proved effective in a retrospective study performed by Tang et al [9]. The aim of this retrospective study was to reevaluate the efficacy of MAHBI in detecting AC.

## Materials and methods

### Demographic and clinical data

The selection criteria were all patients who underwent surgery with a clinical diagnosis of acute cholecystitis from January 2010 to September 2011. They all presented with epigastric or right-upper quadrant abdominal pain and were sent for hepatobiliary imaging. The post-morphine part was determined later by nuclear medicine physicians. There were 68 patients (36 male and 32 female, age = 54 ± 17 years). All patients underwent surgery within one week of MAHBI. Their images were analyzed visually and histopathologic results of gallbladder were retrospectively reviewed. This study has been reviewed and approved by the Human Investigation Committee. Exclusion criteria were (a) Scans were performed to evaluate a disease other than AC. (b) No pathological proof. (c) No post-morphine part. (d) Previous cholecystectomy. (e) Acute pancreatitis. (f) Alcoholism. (g) Sphincterotomy. (h) Taking chronic opiate pain medications. (i) Total parenteral nutrition. Thirty-eight patients were excluded because they did not receive surgery after MAHBI. Two patients were excluded due to acute pancreatitis.

### Imaging protocol

**Fig. 1:**
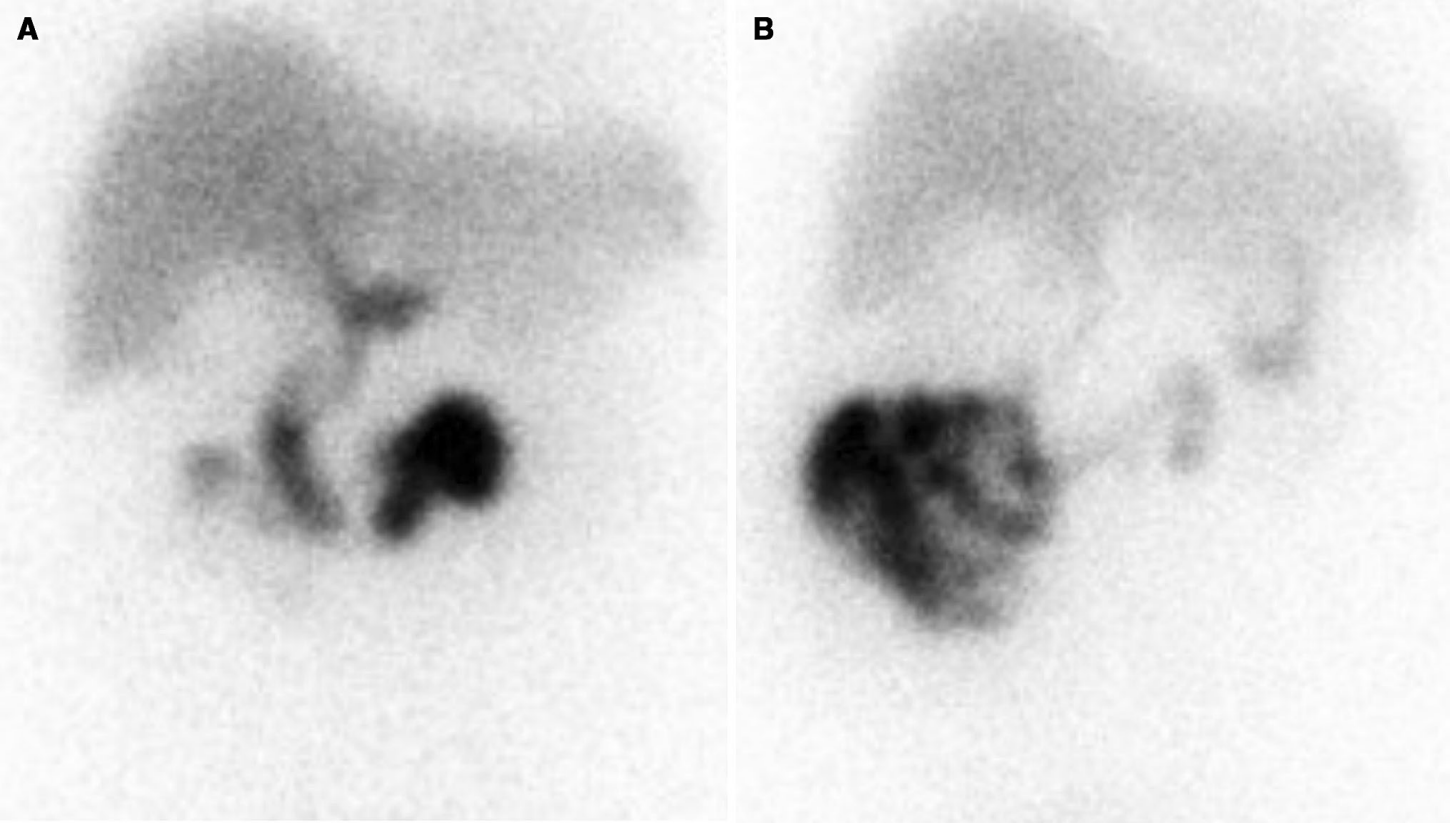
Representative true positive morphine-augmented hepatobiliary imaging. **A** Hepatobiliary imaging at 60 min after radiotracer injection. There was no gallbladder visualization after passage of the radiotracer into the common bile duct and small bowel. **B** Hepatobiliary imaging at 30 min after morphine administration. Even 30 min after morphine administration, the gallbladder was not seen, indicating acute cholecystitis. The pathological finding was consistent with acute cholecystitis

MAHBI was performed 4–6 h after fasting with 185 MBq (5 mCi) of technetium-99 m disofenin using a large-field-of-view γ-camera fitted with a low-energy, parallel-hole collimator. Anterior view hepatic phase dynamic images were obtained for 30 min followed by anterior static images at 45, 50, 55, and 60 min post-radiotracer injection and gallbladder phase dynamic images were obtained for an additional 30 min. The total duration of imaging was 90 min. Both phase images were acquired separately and recorded on a 128 × 128 × 16 computer matrix. After nonvisualization of gallbladder at 60 min had been confirmed, a booster dose of 74–111 MBq (2–3 mCi) technetium-99 m disofenin was injected. This was followed by the injection of 0.04 mg of morphine sulfate per kilogram for 3 min provided that radiotracer was seen within the small bowel. Cholecystokinin was not used in this examination because it might induce gallbladder contraction against a contracted sphincter of Oddi, increasing patient discomfort (Fig. 1).

### Imaging interpretation

**Fig. 2:**
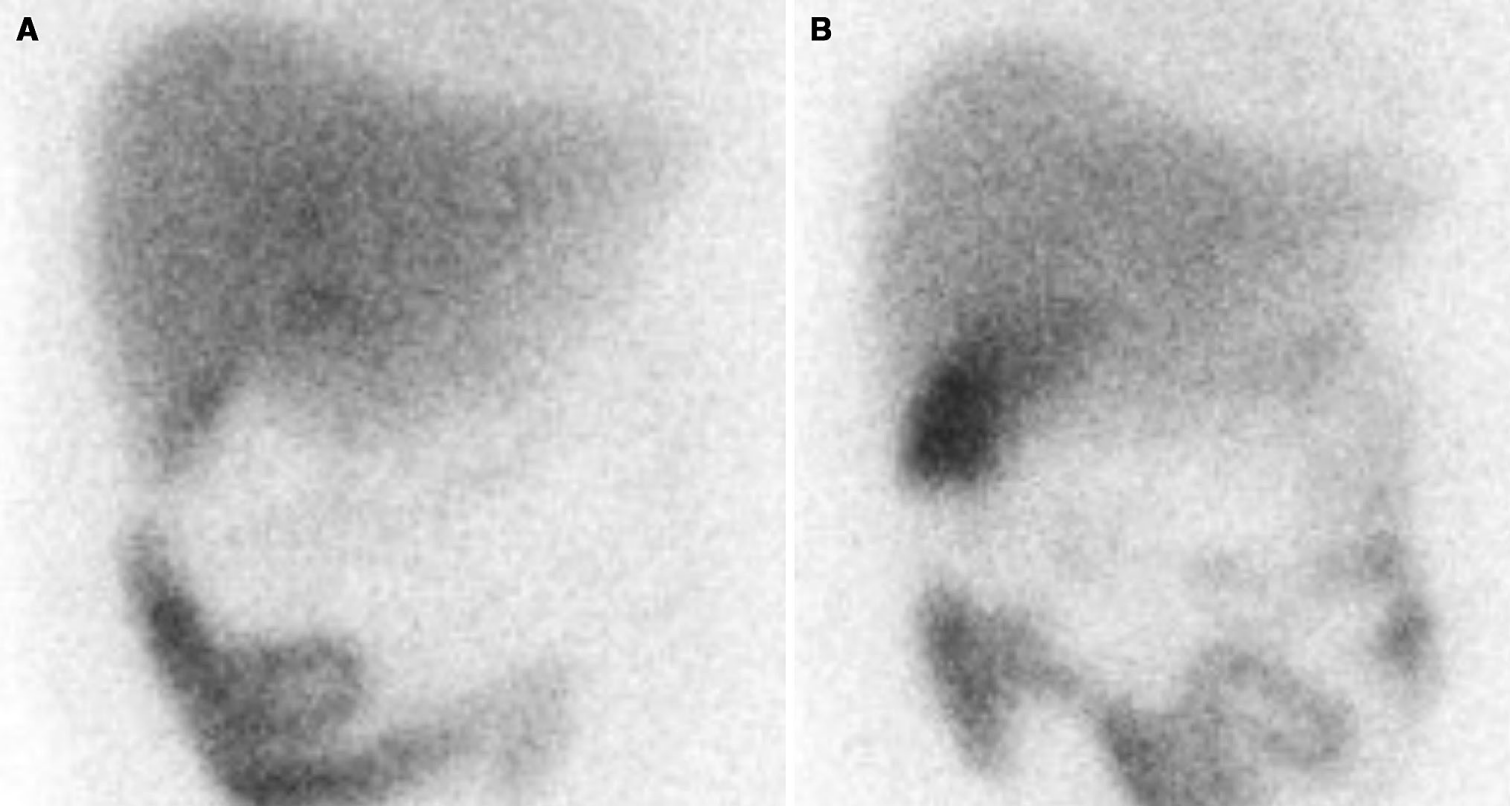
Representative true negative morphine-augmented hepatobiliary imaging. **A** Hepatobiliary imaging at 60 min after radiotracer injection. There was no definite gallbladder visualization after passage of the radiotracer into the small bowel. **B** Hepatobiliary imaging at 30 min after morphine administration. At 30 min after morphine administration, the gallbladder was clearly seen, excluding acute cholecystitis. The pathological finding was chronic cholecystitis

To avoid interobserver variability, all images were interpreted carefully by two experienced board-certified nuclear medicine physicians without knowledge of the clinical or histopathologic findings. If two readers’ interpretations were conflicting, a third nuclear medicine physician reviewed the study. Nonvisualization of gallbladder by 30-min post-morphine administration was considered as a positive MAHBI for AC. The results of image analysis were finally correlated with the gold standard of pathological findings (Fig. 2).

### Statistical analysis

Using pathological gold standard, descriptive statistics were used to describe the basic features of the data in the study. The chi-square test or the Fisher’s exact test was used to evaluate associations between categorical variables. A *p*-value less than 0.05 was considered significant in all tests.

## Results

Of 68 patients with surgery, 45 patients had AC as defined by neutrophilic infiltration, necrosis, hemorrhage, or edema, 21 patients had CC as defined by lymphocytic and plasma cell infiltration, fibrosis, or wall thickening, and two patients had adenocarcinoma of gallbladder. If pathologic features of AC and CC were both seen, the patient was classified as AC in this study. Of 45 patients with AC, 13 (13/45, 29%) patients were AAC and 32 (32/45, 71%) patients were ACC. Of 21 patients with CC, only one (1/21, 5%) patient was CAC and the remaining 20 (20/21, 95%) patients were CCC. Gallstones were also seen in two patients with adenocarcinoma of gallbladder. Significant correlation of AC and MAHBI was observed (*p* < 0.05). As shown in Table 1, there were 45 TP, 19 FP, 4 TN, and no FN cases using gallbladder visualization by 30-min post-morphine infusion as the criteria. High false positive rate of 83% (19/23) was observed. The sensitivity, specificity, accuracy, positive, and negative predictive values of MAHBI in detecting AC was 100%, 17%, 72%, 70%, and 100%, respectively. Gallstones were presented in all FP cases (19/19, 100%), while 32 TP cases (32/45, 71%) were found to have gallstones. Gallstones were seen more frequently in FP cases than TP cases (100% vs. 71%, *p* < 0.05).

**Table 1:** Results of morphine-augmented hepatobiliary imaging

Histopathology	Visualization of gallbladder within 30-min post-morphine infusion	Nonvisualization of gallbladder within 30-min post-morphine infusion
Acute cholecystitis	0	45 (32 with gallstones)
Chronic cholecystitis or gallbladder cancer	4 (3 with gallstones)	19 (19 with gallstones)

## Discussion

The reported sensitivity of MAHBI was consistently high [2, 5–7, 9, 10]; however, some reported specificity was not as good as sensitivity due to the relatively high false positive rate [5, 8–10]. However, it is important to keep in mind that different variations of MAHBI techniques and different percentage of pathologic-confirmed AC were included into the analyses, which is statistically suboptimal. In the current study of 68 patients with histopathologic proof, the sensitivity, specificity, and accuracy were 100%, 17%, and 72%, respectively. Although there was perfect sensitivity, the specificity was low. However, it is likely that the specificity of this examination has been overestimated by previous researchers, influencing current operative decisions.

The current results demonstrated considerably low specificity of MAHBI. The exact reason is not very clear; however, selection bias may play a role in this respect. In other words, the determination of operation by surgeons will affect the study results. In most centers where this study was performed, surgeons tended to select patients with positive MAHBI for operation, and chose conservative treatment for those with negative MAHBI. As a consequence, there might be less true negative MAHBI and more false positive MAHBI detected. Fig et al [10] reported a 69% specificity of MAHBI in patients with severe illnesses, indicating occurrence of high positive rate in patients with severe illnesses. In this study, 17 FP cases (17/19, 89%) had CC, suggesting that CC is probably the most common cause of FP rate in MAHBI since we did not intentionally include patients with severe illnesses. More specifically, no concurrent severe illnesses were found in current study for all FP cases. However, the result is similar to previously published literatures using hepatobiliary imaging without morphine augmentation [11–13]. Therefore, most false positive results for AC are secondary to CC in hepatobiliary imaging whether morphine is used or not. Previous investigators have also indicated that rapid biliary-to-bowel transit might potentially cause false positive MAHBI because of insufficient radiotracer activity remaining in the liver to permit visualization of gallbladder post-morphine augmentation despite a patent cystic duct [7]. In the current study, each patient received a booster dose of 74–111 MBq (2–3 mCi) technetium-99 m disofenin after nonvisualization of gallbladder at 60 min had been confirmed so that rapid biliary-to-bowel transit would not be a significant issue. Rare cases with potential for false-positive interpretations include congenital gallbladder absence, choledochal cysts, cystic fibrosis, inflammation within the immediate proximity of the gallbladder fossa, rupture of a hydatid cyst into biliary tree, unusually elongated gallbladder that is mistaken for the bowel, primary or secondary gallbladder neoplasms, and ceftriaxone therapy [14].

The differences in methodology contribute greatly to the differences in the reported specificity of MAHBI. It is very important to know whether the final diagnoses were confirmed by surgical pathology. The percentage of pathologic-confirmed AC varies widely. A large majority of previous studies used both pathology results and clinical follow up as final diagnosis, while this study adopted only pathology results as final diagnosis. Like many other diseases, the gold standard for diagnosing AC is pathological examination of the gallbladder. Thus, we believe that the results of the current study are convincing although the patient number operated on the negative MAHBI might be small. Nevertheless, the use of a strict pathological criterion for AC (e.g., transmural inflammatory infiltration) might ignore the natural history of this condition. Cholecystectomy specimen removed for AC often shows telltale evidence of pervious inflammatory episodes of AC, with fibrotic, reparative processes. Such fibrotic, reparative processes most likely hinder the development of acute transmural inflammatory changes, and patients with AC superimposed on CC are mislabeled as CC using such a criterion [15]. Patients with CC are more likely to have gallstones than patients with AC in the current study. Since CC may be the major cause of FP MAHBI, it is not surprising to observe more gallstone disease in FP MAHBI in comparison with TP MAHBI. It is postulated that in patients with CC, there is probably stasis of concentrated thick bile in the diseased gallbladder, mainly the cystic duct that precludes gallbladder visualization. The presence of bile sludge can only be suggested by gallbladder nonvisualization in MAHBI.

Inflammatory gallbladder disease presents as a clinical and pathologic spectrum which ranges from acute to subacute to chronic. Distinctions between these entities often are not clear-cut. AC has been reported to correlate most closely with nonvisualization on hepatobiliary imaging but delayed visualization may also be seen. CC may correlate most closely with delayed visualization but nonvisualization also occurs as we have demonstrated in the current study. Such overlap of diseases and findings can cause confusion. However, there is agreement in the literatures that a positive hepatobiliary imaging (nonvisualization or delayed visualization) correlates closely with cholecystitis (acute, subacute, or chronic). Freitas and Gulati make a sound argument that CC patients should undergo cholecystectomy anyway and differentiation from AC may be a moot point. Hence, a false positive study may not be a deleterious error [12, 14, 16].

## Conclusion

MAHBI is sensitive but may not specific for diagnosing AC due to the potential pitfall of high false positive rate. It is of paramount importance to determine whether local surgical preference is for early surgical intervention or conservative management followed by scheduled elective surgeries. Correlation with physical examination, clinical history, abdominal ultrasound, and laboratory findings is highly recommended for optimal patient management.

## References

[1] Ahmed A, Cheung RC, Keeffe EB (2000). Management of gallstones and their complications. Am Fam Physician.

[2] Choy D, Shi EC, McLean RG (1984). Cholescintigraphy in acute cholecystitis: use of intravenous morphine. Radiology.

[3] Kim EE, Pjura G, Lowry P, Nguyen M, Pollack M (1986). Morphine-augmented cholescintigraphy in the diagnosis of acute cholecystitis. AJR.

[4] Keslar PJ, Turbiner EH (1987). Hepatobiliary imaging and the use of intravenous morphine. Clin Nucl Med.

[5] Vasquez TE, Greenspan G, Evans DG, Halpern SE, Ashburn WL (1988). Clinical efficacy of intravenous morphine administration in hepatobiliary imaging for acute cholecystitis. Clin Nucl Med.

[6] Flancbaum L, Alden SM (1990). Morphine cholescintigraphy. Surg Gynecol Obstet.

[7] Fink-Bennett D, Balon H, Robbins T, Tsai D (1991). Morphine-augmented cholescintigraphy: its efficacy in detecting acute cholecystitis. J Nucl Med.

[8] Kim CK, Tse KK, Juweid M (1993). Cholescintigraphy in the diagnosis of acute cholecystitis: morphine augmentation is superior to delayed imaging. J Nucl Med.

[9] Tang B, Phillips V, Natwa M (2011). Evaluation of scan time post morphine injection for diagnosing acute cholecystitis with hepatobiliary imaging [abstract]. J Nucl Med.

[10] Fig LM, Wahl RL, Stewart RE, Shapiro B (1990). Morphine-augmented hepatobiliary scintigraphy in the severely ill: caution is in order. Radiology.

[11] Suarez CA, Block F, Bernstein D (1980). The role of H.I.D.A./P.I.P.I.D.A. scanning in diagnosing cystic duct obstruction. Ann Surg.

[12] Freitas JE, Gulati RM (1980). Rapid evaluation of acute abdominal pain by hepatobiliary scanning. JAMA.

[13] Worthen NJ, Uszler JM, Funamura JL (1981). Cholecystitis: Prospective evaluation of sonography and 99mTc-HIDA cholescintigraphy. Am J Roentgenol.

[14] Allen TW, Tulchinsky M (2013). Nuclear medicine tests for acute gastrointestinal conditions. Semin Nucl Med.

[15] Freitas JE, Coleman RE, Nagle CE (1983). Influence of scan and pathologic criteria on the specificity of cholescintigraphy: concise communication. J Nucl Med.

[16] Tulchinsky M, Colletti PM, Allen TW (2012). Hepatobiliary scintigraphy in acute cholecystitis. Semin Nucl Med.

